# The Effect of Orthognathic Surgery on the Hyoid Bone Position in Skeletal Class III Patients: An Evaluation Using Cephalometric Analysis

**DOI:** 10.29252/wjps.10.2.46

**Published:** 2021-05

**Authors:** Hojjat Hasanzadeh Moghaddam, Ali Labafchi, Samareh Mortazavi, Maryam Khorasanchi, Elahe Tohidi, Seyed-Hosein Hoseini-zarch, Sahand Samieirad

**Affiliations:** 1 Student Research Committee, Faculty of Dentistry, Mashhad University of Medical Sciences, Mashhad, Iran;; 2 Oral and Maxillofacial Radiology, Mashhad Dental School, Mashhad University of Medical Sciences, Mashhad, Iran;; 3 Associate Professor, Oral and Maxillofacial Diseases Research Center, Mashhad University of Medical Sciences, Mashhad, Iran

**Keywords:** Orthognathic surgery, Hyoid bone, Cephalometric analysis

## Abstract

**BACKGROUND:**

This study aimed to investigate the positional changes of the hyoid bone following orthognathic surgery in skeletal class III patients.

**METHODS:**

This double-blinded quasi-experimental study was carried out in Mashhad University of Medical Sciences, Iran, from Apr 2019 to Jun 2020. All skeletal Class III patients who were candidates for orthogenetic surgery were included. They underwent mandibular setback surgery using bilateral sagittal split osteotomy. Cephalometric assessments in relation to hyoid bone position and posterior airway space (PAS) were conducted one day preoperatively (T0), as well as one week (T1), six months (T2) and one year (T3) postoperatively, determining the parameters like the Long axis of the hyoid bone (LAH), Retrognation (RGn), Hyoidale (H), Palatal plane (PP), Mandibular plane (MP) and C3 Vertebrae (C3). All cephalograms were examined using AudaxCeph, Planmeca software. The data analysis was performed via SPSS-19 (*P*<0.05)

**RESULTS:**

25 class III patients, including 18 females (72%) and 7 males (28%) with a mean age of 24.32±5.87, were studied. The cephalometric analysis demonstrated significant decreases in variable angles during the follow-up periods, except for LAH-MP angle (*P*<0.001). The hyoid bone moved downward and backward relative to its original position following the mandibular setback surgery. However, the bone gradually returned to the preoperative location between 6 to 12 months postoperatively.

**CONCLUSION:**

The findings revealed the downward and backward movement of the hyoid bone following the mandibular setback surgery, returned near to its preoperative position after 1 year, postoperatively.

## INTRODUCTION

Orthognathic surgery for skeletal malformation is the distinguished care and practice for enhancing facial esthetics, dental occlusion, and maxillomandibular relations^1^^-^^3^. However, the review of the literature suggested a possible association between the orthognathic surgeries and changes in posterior airway space (PAS)^4^^-^^7^. The soft palate, tongue, and hyoid bone are attached directly or indirectly to the maxilla and mandible. Therefore, the above-mentioned structures are directly affected by the jaw movement, causing alterations in the pharyngeal area^4^^,^^ 6^^-^^11^.

Mandibular setback surgery with or without maxillary advancement is a common treatment plan in patients with skeletal class III malocclusion^1^^, ^^3^^, ^^12^^,^^13^. 

Mandibular displacement during orthognathic surgery may affect the positioning of the hyoid bone and tongue and therefore contribute to narrowing of the airway and development of obstructive sleep apnea^3^^, ^^12^^-^^18^.

The hyoid bone is an important part of the craniomaxillofacial complex^4^^, ^^6^^-^^11^. The position of the hyoid bone is affected by the supra- and infrahyoid muscles, and by the elastic membranes of the larynx and the trachea^6^^, ^^13^^, ^^19^^, ^^20^. The alterations in mandibular position are related to the hyoid bone changes^4^^-^^11^^, ^^13^^, ^^15^^, ^^18^^-^^21^. Moreover, the hyoid bone position adapts to anteroposterior changes in head posture^6^^, ^^13^^, ^^19^^, ^^20^. Thus, evaluation of the hyoid bone positional changes after mandibular setback surgery in patients with Class III deformity is mandatory. 

Lateral cephalometric radiography remains an important imaging tool in orthognathic surgical planning^6^^, ^^9^^, ^^10^^, ^^14^^, ^^22^^-^^24^. It allows the maxillofacial surgeon to plan the surgery and jaw movements, as well as collect relevant information about the hard and soft tissue structures and airway space^6, 17^. Even though this radiographic imaging provides only two-dimensional images for the evaluation of the pharyngeal airway and hyoid bone, it is still used to evaluate sleep disorders and skeletal deformities^6^^, ^^9^^, ^^10^^, ^^14^^, ^^22^^-^^24^.

To the best of our knowledge, few studies investigated the long-term changes in hyoid bone position following orthognathic surgery^6^^-^^9^^, ^^19^^, ^^21^. However, the long-term effect of mandibular setback surgery on the hyoid bone movement is still a controversial topic^2^^, ^^6^^-^^9^^, ^^11^^, ^^19^^, ^^21^. Hence, regarding the influence of the hyoid bone position on the posterior airway space (PAS), the aim of this study was to investigate the positional changes of hyoid bone following orthognathic surgery in skeletal class III patients for a 12-month follow-up period in the Iranian population, using lateral cephalometric analysis.

## MATERIALS AND METHODS

***Study design and patient selection***

This before-after double-blinded quasi-experimental study was performed in Ghaem Hospital, and the Oral and Maxillofacial Surgery Department of Mashhad Dental School, Mashhad University of Medical Sciences, Mashhad, Iran, from Apr 2019 to Jun 2020.

The inclusion criteria were all the healthy patients who were candidates to treat class III malocclusion. Participants were both male and female, aged from 18 to 40 yr old who were ASA I, II regarding systematic conditions. Patients suffering from respiratory and airway problems were excluded as well as those who were not willing to continue participation or follow-ups.

This experiment consisted of two groups. Setback surgery of mandible (BSSO-Bilateral Sagittal Split Osteotomy technique) was performed in one group (Monomax surgery) and in another group, bimaxillary orthognathic operations (Bimax) including maxillary advancement (LeFort I) plus mandibular setback (BSSO) were done.

The need for mandibular setback plus maxillary advancement was established by clinical and cephalometric examinations in our cases. 

All orthognathic surgeries were conducted by the same surgeon and the same surgical team and hospital. For general hypotensive anesthesia, all patients were given the same standard intravenous drug regimens.

Patients underwent mandibular setback surgery (BSSO) with or without maxillary advancement (LeFort I). The rigid fixation of the maxillomandibular segments was performed. 

***Variables and Data collection***

Age, gender, date, and type of surgery as well as the amount of maxillomandibular displacement were recorded. The change in the position of the hyoid bone was the primary outcome variable in the present study. 

All the patients with Cl III malocclusion who were eligible to enter the study were asked to complete a demographic questionnaire and take a lateral cephalogram one day before the surgery (T0) at Ghaem Hospital. Then positional evaluations of hyoid bone were performed based on the radiographs.

The dependent variables were as follows the Long axis of the hyoid bone (LAH), Retrognation (RGn), Hyoidale (H), Palatal plane (PP), Mandibular plane (MP), and C3 Vertebrae (C3). Postoperative radiographic analyses were done 1 week (T1), six months (T2), and one year (T3) later to reevaluate the position of hyoid bone. All cephalograms were analyzed using AudaxCeph, Planmeca software (Planmeca, Helsinki, Finland).

**Fig. 1:**
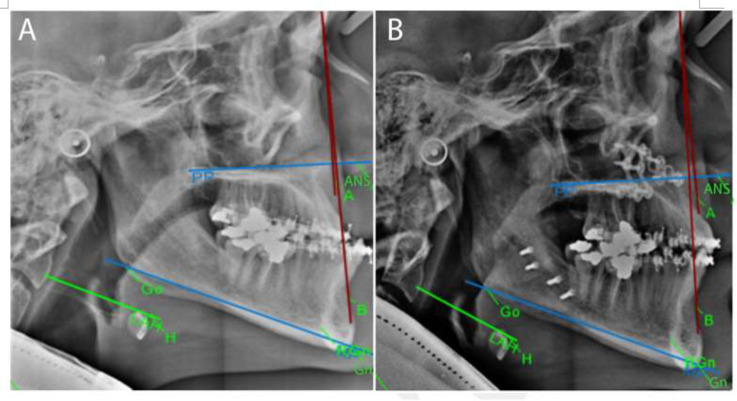
Lateral cephalometric analysis of patient with traced hyoid landmarks: (A) before the surgery (T0) and (B) after 1 year of follow up (T3).

The best approach for this investigation was to use anatomical landmarks adopted from Mortazavi et al.^6^ survey. The use of these landmarks enabled us to evaluate the position of hyoid bone in two dimensions and the sagittal plane comprehensively (Figure 1).

Several parameters were measured in all patients, including the distance between H and MP, H and PP, H and C3, H and RGn as well as angulation between LAH and MP and also between LAH and PP. Other variables are the type of deformity, age, gender, movement of the jaw due to surgery, and the number of jaws involved.

Both the statistical and cephalometric analyst were unaware of the surgical procedure as well as the amount of maxillomandibular displacement (in millimeters). However, the surgeon and the student who had completed the checklist were aware of the surgery.

***Statistical analyses***

Collected data analysis was carried out via repeated measures ANOVA test and SPSS version 19 (SPSS Inc., Chicago, IL). Qualitative variables were expressed as a percentage, while quantitative variables were stated as mean ± SD (standard deviation). *P*-value less than 0.05% was considered significant.

***Ethical Approval***

All procedures performed in this study involving the human participant were in accordance with the ethical standards of our institutional Research Committee and with the 1964 Helsinki declaration. The patient's ethical consent form was signed and approved by the patient. All procedures used in this research were approved by the Ethical Committee of Mashhad University of Medical Sciences (IR.MUMS.DENTISTRY.REC.1398.104).

## RESULTS

There were 25 participants, including 18 females (72%) and seven males (28%) aged from 18 to 37 and with a mean age of 24.32±5.87. One group (n=7) underwent Monomax, and the other group (n=18) treated by the Bimax technique.

Amounts of jaw displacement during the orthognathic surgeries were calculated. The mean amount of the mandibular setback in patients was 5.24 ± 1.48 mm.

The mean of mandibular setback displacement for the group in which the monomax (isolated mandibular setback) surgery was performed, was considerably more than the bimaxillary operated samples (*P*<0.001) (Table 1). Table 2 indicated no significant association between the hyoid parameter alterations and age, mandibular seatback or maxillary advancement.

With respect to comparison between monomaxillary and bimaxillary surgeries, the mean changes regarding angulation of LAH_PP, LAH_MP and mean changes of H_PP distance as well as H_RGn distance in bimaxillary surgeries were less than monomax, with no significant differences (*P*=0.495, *P*=0.547, *P*=0.790 and *P*=0.498, respectively). In fact, the average of changes in H_MP and H_C3 distance were more in the bimaxaillary surgeries compared to isolated mandibular setback operations (Table 3).

Moreover, the average of changes regarding H_PP, H_MP, H_RGn and LAH_PP in the males was less than the females, with no significant difference (Table 4).

**Table 1:** Mandibular displacement amounts in Monomaxillary and Bimaxillary surgery

Surgery	Number (N)	Mean SD	Median	Independent *t* test
**Monomaxillary**	**7**	**7.140.900**	**7.00**	**Z=3.79** ***P*** **<0.001**
**Bimaxillary**	**18**	**4.500.857**	**4.50**	

**Table 2:** The correlation between hyoid variables and age, mandibular setback and maxillary advancement amount

Variables (parameters)		Age (N=25)	Amount of Mandibular setback (N=25)	Amount of Maxillary advancement (N=18)
**H_PP Distance**	**Spearman's correlation coefficient**	**0.046**	**0.219**	**0.158**
	**P-value**	**0.828**	**0.293**	**0.531**
**H_MP Distance**	**Pearson's correlation coefficient **	**0.111**	**-0.137**	**-0.099**
	**P-value**	**0.597**	**0.515**	**0.695**
**H_C3 Distance**	**Pearson's correlation coefficient**	**-0.051**	**-0.088**	**-0.228**
	**P-value**	**0.809**	**0.676**	**0.364**
**H_RGn Distance**	**Pearson's correlation coefficient**	**0.162**	**0.109**	**0.245**
	**P-value**	**0.439**	**0.604**	**0.327**
**LAH_PP Angle**	**Spearman's correlation coefficient**	**0.205**	**-0.192**	**-0.107**
	**P-value**	**0.325**	**0.357**	**0.672**
**LAH_MP Angle**	**Spearman's correlation coefficient**	**-0.173**	**0.160**	**0.146**
	**P-value**	**0.407**	**0.445**	**0.563**

**Table 3:** Comparison of hyoid parameters between the Monomaxillary and Bimaxillary surgery

Variables (Parameters)	Surgery	Number (N)	Mean SD	Median	*P*-value
**H_PP Distance**	**Monomaxillary**	**7**	**0.335.85**	**-.85**	**Z=0.27** ***P*** **=0.790**
	**Bimaxillary**	**18**	**-0.493.94**	**-1.22**	
**H_MP Distance**	**Monomaxillary**	**7**	**21.4820.99**	**21.88**	**T=0.28** ***P*** **=0.785**
	**Bimaxillary**	**18**	**23.3212.16**	**22.69**	
**H_C3 Distance**	**Monomaxillary**	**7**	**1.173.21**	**2.38**	**T=0.04** ***P*** **=0.971**
	**Bimaxillary**	**18**	**1.234.14**	**1.18**	
**H_RGn Distance**	**Monomaxillary**	**7**	**12.972.75**	**13.10**	**T=0.69** ***P*** **=0.498**
	**Bimaxillary**	**18**	**11.385.80**	**10.78**	
**LAH_PP Angle**	**Monomaxillary**	**7**	**-3.2216.45**	**-10.71**	**Z=0.70** ***P*** **=0.495**
	**Bimaxillary**	**18**	**-3.9214.68**	**-3.00**	
**LAH_MP Angle**	**Monomaxillary**	**7**	**-22.77102.36**	**-10.00**	**Z=0.58** ***P*** **=0.574**
	**Bimaxillary**	**18**	**-48.4299.69**	**-14.14**	


Z: Mann Whitney Test

T: T-test

Nevertheless, the average of LAH_MP angulation changes in the males was significantly more than females (*P*=0.027) (Table 4).

Comparison of the hyoid variables during the time intervals of follow-ups demonstrated the significant changes in parameters after the orthognathic surgery except for LAH_MP (*P*<0.001) (Table 5).

 The mean distance of H_PP at T1 was significantly higher than at other time intervals (*P*<0.001) and at T2 was significantly higher than T0, as well (*P*<0.001). The mean H-MP, H-C3, and the mean distance of H_RGn were significantly decreased at all-time intervals compared to T0 (*P*<0.001). Moreover, these parameters were significantly reduced at T3 compared to T1 (*P*<0.001).The mean angle of LAH_PP at T1 was significantly lower than T0. In addition, the mean angle of LAH_PP at T3 was significantly less than T1 and T2 (*P*<0.001). There was no significant difference between other two-pair comparisons for any of the variables (Table 5).

**Table 4:** Comparison of hyoid parameters between the males and females

Variables (Parameters)	Gender	Number (N)	Mean SD	Independent T Test
**H_PP Distance**	**Female**	**18**	**0.465.00**	**T=1.33** ***P*** **=0.061**
	**Male**	**7**	**-2.121.50**	
**H_MP Distance**	**Female**	**18**	**23.7411.76**	**T=0.50** ***P*** **=0.622**
	**Male**	**7**	**20.4221.48**	
**H_C3 Distance**	**Female**	**18**	**0.383.51**	**T=1.82** ***P*** **=0.082**
	**Male**	**7**	**3.354.07**	
**H_RGn Distance**	**Female**	**18**	**12.384.71**	**T=0.87** ***P*** **=0.395**
	**Male**	**7**	**10.396.24**	
**LAH_PP Angle**	**Female**	**18**	**-0.9211.39**	**T=1.56** ***P*** **=0.133**
	**Male**	**7**	**-10.9320.74**	
**LAH_MP Angle**	**Female**	**18**	**-60.32109.77**	**T=1.59** ***P*** =0.027*
	**Male**	**7**	**7.8233.84**	


*: Significant difference.

## DISCUSSION

Our study findings revealed the downward and backward movement of the hyoid bone following the mandibular setback surgery, returned near to its preoperative position after one year, postoperatively. 

Few researchers have studied the alterations in the position of hyoid bone due to orthognathic surgeries and there are still some controversies within the literature concerning this issue^2^^, ^^6^^-^^9^^, ^^11^^, ^^19^^, ^^21^. Regarding the published articles, after mandibular setback surgery the hyoid bone may move inferiorly, both inferiorly and posteriorly, and both inferiorly and forward^7^^-^^9^^, ^^11^^, ^^15^^, ^^17^^-^^21^^, ^^24^^, ^^25^. The hyoid bone returns to its original position, maintaining the airway resistance^4^^, ^^6^^, ^^7^^, ^^12^^, ^^19^^, ^^26^. These changes can occur because of the physiologic reflex mechanism for maintaining the posterior airway space^4^^, ^^6^^, ^^7^^, ^^12^.

In orthognathic surgeries, the hyoid bone acts as a reference for measuring and evaluating the position of the mandible and maxilla^6^^, ^^13^^, ^^19^^, ^^20^. The hyoid bone is considered as the base of the tongue due to the close relationship between these structures^6^^, ^^13^^, ^^19^^, ^^20^. Therefore, the position of the hyoid bone plays a crucial role in obtaining the posterior airway space. The mandibular setback surgery (BSSO) involves the changes regarding the hyoid position and as a result, affects the posterior airway space^4^^-^^11^^, ^^13^^, ^^15^^, ^^18^^-^^21^. Mandibular displacement during orthognathic surgery may affect the positioning of the hyoid bone and tongue and therefore contribute to narrowing of the airway and development of obstructive sleep apnea^6^^, ^^13^^, ^^19^^, ^^20^.

Cephalometric assessments are valuable for determination of the hyoid bone position and posterior airway space (PAS) due to recent studies^2^^, ^^6^^-^^9^^, ^^11^^, ^^19^^, ^^21^.

**Table 5:** The changes of hyoid parameters following the orthognathic surgery in different time intervals

Variables (Parameters)	Intervals	Number (N)	MEAN * SD	Repeated Measures ANOVA Test
**H_PP Distance**	**T0**	**25**	**56.70** ^a^ **6.65**	***P*** <0.001*
	**T1**	**25**	**61.56** ^b^ **6.40**	
	**T2**	**25**	**57.68** ^c^ **6.87**	
	**T3**	**25**	**56.86** ^a,c^ **7.31**	
**H_MP Distance**	**T0**	**25**	**12.26** ^a^ **5.40**	***P*** <0.001*
	**T1**	**25**	**10.52** ^b^ **5.38**	
	**T2**	**25**	**10.42** ^b,c^ **5.53**	
	**T3**	**25**	**9.50** ^c^ **4.65**	
**H_C3 Distance**	**T0**	**25**	**38.16** ^a^ **5.71**	***P*** <0.001*
	**T1**	**25**	**34.46** ^b^ **5.69**	
	**T2**	**25**	**37.24** ^c^ **5.49**	
	**T3**	**25**	**37.70** ^a,c^ **5.75**	
**H_RGn Distance**	**T0**	**25**	**38.60** ^a^ **6.05**	***P*** <0.001*
	**T1**	**25**	**35.16** ^b^ **6.24**	
	**T2**	**25**	**34.60** ^b^ **6.72**	
	**T3**	**25**	**34.08** ^c^ **6.06**	
**LAH_PP Angle**	**T0**	**25**	**24.90** ^a,c^ **7.69**	***P*** <0.001*
	**T1**	**25**	**22.64** ^b^ **9.21**	
	**T2**	**25**	**24.24** ^a,b^ **8.89**	
	**T3**	**25**	**25.88** ^c^ **8.58**	
**LAH_MP Angle**	**T0**	**25**	**4.645.59**	***P*** **=0.228**
	**T1**	**25**	**5.665.33**	
	**T2**	**25**	**5.465.46**	
	**T3**	**25**	**5.684.65**	

**T0:** **one day before the surgery.**

** T1: 1 week, T2: six months, and T3: one year postoperatively.**

*: Significant difference.

The superscript letters had significant relations.

The hyoid bone moves downward and backward relative to the previous position following the mandibular setback surgery. Similarly, back and downward reposition were reported^15^^,^^21^, whereas no significant change in the location of the hyoid bone was observed in other studies^19^^,^^26^. 

In contrast to our findings, Tselnik et al reported forward and downward movement of hyoid bone immediately after the setback surgery^27^, which may be due to different criteria of measurement and the effect of maxillary impaction on the occlusal plane and hyoid plane alteration, consequently^1^^, ^^6^.

Accordingly, an upward movement of hyoid bone was reported following the bimaxillary surgery^20^. Moving toward upper position is a physiological adaptation to sustain the airway opening. The controversies regarding the results of previous studies may be due to the difference in sample sizes, cephalometric analysis parameters, and simultaneous maxillary vertical plane movements in these studies^1^^, ^^4^^, ^^6^^, ^^17^. 

Furthermore, in our study, the hyoid bone gradually returned to the preoperative position in 6 to 12 months follow-up intervals, observed in both Monomax and Bimax surgeries. This finding was consistent with the prior study results^3^^, ^^6^^, ^^8^^, ^^9^^, ^^19^^, ^^21^. As proposed, a study of 46 Cl III samples was performed, of which 25 participants underwent the bilateral sagittal split osteotomy (BSSO), and 21 subjects received bilateral intraoral vertical ramus osteotomy (IVRO)^10^. The hyoid bone showed a tendency to return to its original place, however, the posterior airway space remains narrow during the follow-up period^10^. 

The sample size of the present study was similar to Efendiyeva et al.^20^, and Kim et al.^28^ study (25 participants), whereas this study included more subjects than Liukkonen et al.^26^ and Foltan et al.^15^, which were 22 and 21 patients, respectively.

The findings of the present study demonstrated no difference related to gender in positional changes of hyoid bone which implied the similar adaptation process of hyoid in male and female patients after the jaw movements in orthognathic surgery. Our result was in line with most of the studies ^3^^, ^^6^^, ^^8^^, ^^9^^, ^^19^^, ^^21^. Nonetheless, the hyoid repositioning happened only in females and reported no significant movement in males^17^.

Despite the movement of the hyoid bone toward the previous position between 6 to 12 months after the operation, it probably might never reach to its former exact location^6^^, ^^7^^, ^^9^^, ^^11^^, ^^26^, which may result in the posterior airway space reduction and post-operative obstructive sleep apnea^1^^,^^3^^, ^^6^^, ^^7^^, ^^9^^, ^^11^^, ^^26^, the bimaxillary surgery was preferred rather than the isolated mandibular setback in the case of skeletal cl III deformity to prevent probable PAS reduction^14^. 

A remarkable feature of this study was its one-year follow-up period which is the same as other studies^8^^,^^9^^, ^^19^^,^^21^. Long-term follow-up allowed us to assure the patients of the impermanency of probable postoperative mentioned complications. Nonetheless, the follow up durations of various studies by^15^^,^^18^^,^^28^ were shorter than the current study.

## CONCLUSION

This study findings revealed the downward and backward movement of the hyoid bone following the mandibular setback surgery, returned near to its normal position after one year, postoperatively. Since fewer changes were observed in the patients underwent Bimax, the bimaxillary surgeries might be advantageous to maintain PAS in cases who required large amounts of mandibular setback reposition. 

## LIMITATIONS

With respect to the sample size of similar research and the pilot status of this study, one limitation of our implementation is the small sample size. Due to the COVID-19 pandemic in the world and Iran and quarantine protocols, it was not possible to follow up more patients. However, the present study statistical results were satisfactory. Future surveys involving a larger sample size are suggested. 

## FUNDING

None. This study was self-funded

## References

[1] Eshghpour M, Mianbandi V, Samieirad S (2018). Intra- and Postoperative Complications of Le Fort I Maxillary Osteotomy. J Craniofac Surg.

[2] Fernández-Ferrer L, Montiel-Company JM, Pinho T, Almerich-Silla JM, Bellot-Arcís C (2015). Effects of mandibular setback surgery on upper airway dimensions and their influence on obstructive sleep apnoea - a systematic review. J Craniomaxillofac Surg.

[3] Yavari N, Samieirad S, Labafchi A, Rezaeetalab F, Eshghpour M (2020). Is There an Increase in the Risk of Obstructive Sleep Apnea After Isolated Mandibular Setback Surgery? An Evaluation Using the STOP-BANG Questionnaire. J Oral Maxillofac Surg.

[4] Hasebe D, Kobayashi T, Hasegawa M, Iwamoto T, Kato K, Izumi N, Takata Y, Saito C (2011). Changes in oropharyngeal airway and respiratory function during sleep after orthognathic surgery in patients with mandibular prognathism. Int J Oral Maxillofac Surg.

[5] Mattos CT, Vilani GN, Sant'Anna EF, Ruellas AC, Maia LC (2011). Effects of orthognathic surgery on oropharyngeal airway: a meta-analysis. Int J Oral Maxillofac Surg.

[6] Mortazavi S, Asghari-Moghaddam H, Dehghani M, Aboutorabzade M, Yaloodbardan B, Tohidi E, Hoseini-Zarch SH (2018). Hyoid bone position in different facial skeletal patterns. J Clin Exp Dent.

[7] Shin JH, Kim MA, Park IY, Park YH (2015). A 2-year follow-up of changes after bimaxillary surgery in patients with mandibular prognathism: 3-dimensional analysis of pharyngeal airway volume and hyoid bone position. J Oral Maxillofac Surg.

[8] Güven O, Saraçoğlu U (2005). Changes in pharyngeal airway space and hyoid bone positions after body ostectomies and sagittal split ramus osteotomies. J Craniofac Surg.

[9] Kawakami M, Yamamoto K, Fujimoto M, Ohgi K, Inoue M, Kirita T (2005). Changes in tongue and hyoid positions, and posterior airway space following mandibular setback surgery. J Craniomaxillofac Surg.

[10] Kitahara T, Hoshino Y, Maruyama K, In E, Takahashi I (2010). Changes in the pharyngeal airway space and hyoid bone position after mandibular setback surgery for skeletal Class III jaw deformity in Japanese women. Am J Orthod Dentofacial Orthop.

[11] On SW, Han MW, Hwang DY, Song SI (2015). Retrospective study on change in pharyngeal airway space and hyoid bone position after mandibular setback surgery. J Korean Assoc Oral Maxillofac Surg.

[12] Canellas JV, Barros HL, Medeiros PJ, Ritto FG (2016). Sleep-disordered breathing following mandibular setback: a systematic review of the literature. Sleep Breath.

[13] Kawamata A, Fujishita M, Ariji Y, Ariji E (2000). Three-dimensional computed tomographic evaluation of morphologic airway changes after mandibular setback osteotomy for prognathism. Oral Surg Oral Med Oral Pathol Oral Radiol Endod.

[14] Demetriades N, Chang DJ, Laskarides C, Papageorge M (2010). Effects of mandibular retropositioning, with or without maxillary advancement, on the oro-naso-pharyngeal airway and development of sleep-related breathing disorders. J Oral Maxillofac Surg.

[15] Foltán R, Hoffmannová J, Donev F, Vlk M, Sedý J, Kufa R, Bulik O (2009). The impact of Le Fort I advancement and bilateral sagittal split osteotomy setback on ventilation during sleep. Int J Oral Maxillofac Surg.

[16] Kitagawara K, Kobayashi T, Goto H, Yokobayashi T, Kitamura N, Saito C (2008). Effects of mandibular setback surgery on oropharyngeal airway and arterial oxygen saturation. Int J Oral Maxillofac Surg.

[17] Samman N, Tang SS, Xia J (2002). Cephalometric study of the upper airway in surgically corrected class III skeletal deformity. Int J Adult Orthodon Orthognath Surg.

[18] Setoudehmaram S, Masoumi S, Danaei SM, Zamiri B (2017). Assessment of Changes in the Hyoid Bone Position Following Orthognathic Surgery in Class III Patients. Avicenna J Dent Res.

[19] Aydemir H, Memikoğlu U, Karasu H (2012). Pharyngeal airway space, hyoid bone position and head posture after orthognathic surgery in Class III patients. Angle Orthod.

[20] Efendiyeva R, Aydemir H, Karasu H, Toygar-Memikoğlu U (2014). Pharyngeal airway space, hyoid bone position, and head posture after bimaxillary orthognathic surgery in Class III patients: long-term evaluation. Angle Orthod.

[21] Marşan G, Oztaş E, Cura N, Kuvat SV, Emekli U (2010). Changes in head posture and hyoid bone position in Turkish Class III patients after mandibular setback surgery. J Craniomaxillofac Surg.

[22] Gokce SM, Gorgulu S, Gokce HS, Bengi AO, Karacayli U, Ors F (2014). Evaluation of pharyngeal airway space changes after bimaxillary orthognathic surgery with a 3-dimensional simulation and modeling program. Am J Orthod Dentofacial Orthop.

[23] Gonçales ES, Rocha JF, Gonçales AG, Yaedú RY, Sant'Ana E (2014). Computerized cephalometric study of the pharyngeal airway space in patients submitted to orthognathic surgery. J Maxillofac Oral Surg.

[24] Muto T, Takeda S, Kanazawa M, Yamazaki A, Fujiwara Y, Mizoguchi I (2002). The effect of head posture on the pharyngeal airway space (PAS). Int J Oral Maxillofac Surg.

[25] Muto T, Yamazaki A, Takeda S, Kawakami J, Tsuji Y, Shibata T, Mizoguchi I (2006). Relationship between the pharyngeal airway space and craniofacial morphology, taking into account head posture. Int J Oral Maxillofac Surg.

[26] Liukkonen M, Vähätalo K, Peltomäki T, Tiekso J, Happonen RP (2002). Effect of mandibular setback surgery on the posterior airway size. Int J Adult Orthodon Orthognath Surg.

[27] Tselnik M, Pogrel MA (2000). Assessment of the pharyngeal airway space after mandibular setback surgery. J Oral Maxillofac Surg.

[28] Kim MA, Kim BR, Choi JY, Youn JK, Kim YJ, Park YH (2013). Three-dimensional changes of the hyoid bone and airway volumes related to its relationship with horizontal anatomic planes after bimaxillary surgery in skeletal Class III patients. Angle Orthod.

